# “I’m not going to lay back and watch somebody die”: A qualitative study of how people who use drugs’ naloxone experiences are shaped by rural risk environment and naloxone distribution/overdose education intervention

**DOI:** 10.21203/rs.3.rs-3310319/v1

**Published:** 2023-09-06

**Authors:** Zora Kesich, Umedjon Ibragimov, Kelli Komro, Kenneth Lane, Melvin Livingston, April Young, Hannah Cooper

**Affiliations:** Emory University Rollins School of Public Health; Emory University Rollins School of Public Health; Emory University Rollins School of Public Health; University of Kentucky College of Public Health; Emory University Rollins School of Public Health; University of Kentucky College of Public Health; Emory University Rollins School of Public Health

**Keywords:** Rural, Naloxone, Narcan, Rural Risk Environment

## Abstract

**Background:**

Overdoses have surged in rural areas in the U.S. and globally for years, but harm reduction interventions have lagged. Overdose education and naloxone distribution (OEND) programs are highly effective to prevent overdose mortality, but little is known about people who use drugs’ (PWUD) experience with these interventions in rural areas. Here, we analyze qualitative data with rural PWUD to learn about their experiences with an OEND intervention, and about how their perceptions of their rural risk environments influenced the interventions’ effects.

**Methods:**

Twenty-nine one-on-one, semi-structured qualitative interviews were conducted with rural PWUD engaged in the CARE2HOPE OEND intervention in Appalachian Kentucky. Interviews were conducted via Zoom, audio-recorded, and transcribed verbatim. Thematic analysis was conducted, guided by the Rural Risk Environment Framework.

**Results:**

The OEND intervention transformed participants’ roles locally, so they became an essential component of the local rural healthcare environment. The intervention provided access to naloxone and information, thereby increasing PWUD’s confidence in naloxone administration. Through the intervention, over half of participants gained knowledge on naloxone (access points, administration technique) and on the criminal-legal environment as it pertained to naloxone. Most participants opted to accept and carry naloxone, citing factors related to the social environment (sense of responsibility to their community) and physical/healthcare environments (high overdose prevalence, suboptimal emergency response systems). Over half of participants described recent experiences administering intervention-provided naloxone. These experiences were shaped by features of the local rural social environment (anticipated negative reaction from recipients, prior naloxone conversations).

**Conclusions:**

By providing naloxone paired with non-stigmatizing health and policy information, the OEND intervention offered the material and informational support that allowed participants to become a part of the healthcare environment. Findings highlight need for more outreach to rural PWUD on local policy that impacts them; tailored strategies to help rural PWUD engage in productive dialogue with peers about naloxone and navigate interpersonal conflict associated with overdose reversal; and opportunities for rural PWUD to formally participate in emergency response systems as peer overdose responders.

**Trial registration:**

The ClinicalTrials.gov ID for the CARE2HOPE intervention is NCT04134767. The registration date was October 19^th^, 2019.

## Background

Overdose burden is elevated in the United States’ rural communities (1), including Appalachian Kentucky (2,3). In 2021, Kentucky had the fifth-highest age-adjusted fatal overdose rate in the U.S., at 1.7 times the national average (4). Naloxone is a highly effective medication to reverse overdose when correctly administered (5–7). Because people who use drugs (PWUD) are among the most likely to witness overdose onset, it is critical that they can access naloxone and are trained in its administration (8,9). Despite the high overdose burden in rural communities and evidence-based recommendations to provide overdose education and naloxone distribution (OEND) to PWUD (10), there are numerous barriers to accessing and utilizing naloxone in rural communities (11–14).

Much of what we know about how PWUD perceive and engage with naloxone comes from studies in urban and suburban areas (3,15–17). This research indicates that social *barriers* to naloxone administration in urban and suburban areas include fear that the recipient will react aggressively upon revival, fear of disrupting someone’s high, and fear of negatively impacting the relationship with the recipient (18, 8–9). Regarding social *facilitators* to naloxone administration, the person administering naloxone can experience a sense of empowerment or pride about saving a life and contributing to the safety of their community (8,17,19). In areas where free/low-cost naloxone is inaccessible, the cost of the drug acts as an *economic* barrier (16). *Criminal-legal* barriers include fear of legal repercussions and police harassment particularly in the aftermath of naloxone administration (16,18). This concern is especially relevant to people with records of criminal-legal involvement, and among Black communities, who have been disproportionately harmed by the United States’ war on drugs (17). Finally, this non-rural research suggests that *healthcare* barriers are rooted in access to instrumental support (e.g., naloxone) and informational support (e.g., education, training) (8,20).

While these studies provide valuable insight, there remain several areas for growth in this body of research. First, there is a paucity of research exploring PWUDs’ first-hand naloxone experiences in rural settings, despite the high overdose burden these areas experience. One study based in rural Alaska found that PWUD had positive feelings towards naloxone and perceived it to be highly effective (21). A study based in rural West Virginia found that some PWUD experienced emotional distress during and after using naloxone to reverse peers’ overdose due to witnessing a near-death event and negative reactions from community members who witnessed the occurrence (18).

Most studies focus on the overdose event itself, with limited research exploring the surrounding environmental context, including PWUD’ decisions to carry or not carry naloxone, how PWUD develop plans governing naloxone administration with one another before an overdose occurs, or how they discuss it with other PWUD after overdose events. Understanding how PWUD perceive and experience naloxone within the context of their environment is critical to addressing and reducing barriers to peer naloxone administration, thereby decreasing rates of fatal overdose.

One approach to exploring environmental features of drug-related harm is Rhodes’ ***Risk Environment Framework (REF)***, which invites us to analyze how complex interplays among individuals and their *economic, physical, social, and political environments* shape the risk of drug-related harms, including overdose (5,22). The REF has evolved over time, and now includes the *healthcare* and *criminal-legal* intervention environments, and recognizes that rural risk environments may be qualitatively different from urban risk environments (11–13, 23).

The current analysis builds on this work by using the ***rural REF (R-REF)*** to qualitatively assesses: 1) *What are the perceived pathways through which risk environments influence whether and how PWUD accept, carry, and administer naloxone?*, and 2) *What is the role of an OEND intervention in modifying these pathways?*

## Methods

### Overview & design

All participants in this sample were part of a parent study, **CARE2HOPE (C2H)**, which assessed the extent to which PWUD benefited from a healthcare navigation, HIV/HCV testing, and OEND intervention. C2H encompasses 12 rural counties in the heart of Appalachian Kentucky’s opioid crisis; six counties were randomized to the intervention condition. Intervention components and the target population were selected by eight community-academic partnership groups (CAPs) that spanned these 12 counties. The target population selected were individuals who were involved in the criminal-legal system. The intervention’s primary targets were reductions in the frequency of illegalized drug use; secondary targets included reductions in overdose. Intervention components included OEND; and Project START (24), a CDC evidence-based initiative designed to reduce HIV and STIs among criminal-legal involved populations who use drugs via motivational interviewing to identify harm reduction goals, develop and implement strategies to meet those goals, and use health navigation methods to connect individuals to needed services; and HIV and HCV counseling, testing, and linkage to care. The OEND component, hereafter referred to as the “C2H OEND intervention”, included overdose prevention education; naloxone and fentanyl test strip distribution; and education about state laws governing naloxone possession and administration and overdose response. C2H intervention sessions and healthcare navigation services were delivered by project staff called “Rural Health Navigators” or “REHNs” who were residents of the communities they served.

This study utilized a qualitative design that used in-depth, semi-structured interviews to explore participant perceptions of the pathways through which the intervention shaped intervention targets. The current analysis focuses on data relevant to perceptions of the OEND component of the intervention.

### Sample & recruitment

Individuals were eligible for C2H if they lived in one of the Kentucky counties targeted by the study; were 18 years old or older; had been engaged in the criminal-legal system in the past month; and either used opioids to get high or injected drugs to get high 30 days prior to criminal legal system involvement. Individuals were recruited into C2H via multiple community-based pathways, including via tabling outside probation and parole offices, courthouses, and harm reduction programs; cookouts near places where eligible individuals might live or seek services; flyers; and word of mouth.

To be eligible for this qualitative study, individuals had to have been enrolled in the C2H intervention for at least three months prior to the interview and to have taken part in the baseline intervention. We (ZK, LP) purposively sampled C2H participants, seeking to create a qualitative sample of people that varied by county and gender. We invited individuals to take part in a qualitative interview through the means of communication they permitted staff to use (phone calls, texts, emails, and/or Facebook messages). Some recruitment was facilitated through C2H REHNs who helped qualitative study staff contact hard-to-reach participants. See Table 1 for a description of the sample.

### Data Collection

In-depth, semi-structured interviews were conducted with all participants between March 18^th^, 2022, and October 24^th^, 2022 ($30 honorarium, provided via a Western Union card). Interviews took place over a HIPAA-protected Zoom interface and lasted between 30 and 94 minutes. Participants without access to private space and/or Zoom-equipped technology utilized C2H office space; REHNs were not in the offices while interviews were conducted.

The interview guide (see Table 2) was informed by literature (including previous research with the study population and other similar populations) 14), theory (including R-REF) (19), and input from C2H REHNs. The guide covered participants’ ***social environments*** (e.g., family support), ***economic environments*** (e.g., financial needs, employment barriers), ***healthcare environments*** (e.g., experience being connected to healthcare services through C2H), ***physical environments*** (e.g., current and prior experiences with homelessness), and ***political/criminal-legal system environments*** (e.g., interactions with law enforcement and criminal justice systems). One domain of interview questions (see Table 3) covered participants’ ***experiences accessing, carrying, and administering naloxone***. Topics in this domain include the participants’ experience accessing, carrying, and administering naloxone prior to their time in the intervention, speaking to research question one: *What are the perceived pathways through which risk environments influence whether and how PWUD accept, carry, and administer naloxone?*; and questions about participants’ experiences accessing, carrying, and administering naloxone during and after the intervention, speaking to research question two: *What is the role of an OEND intervention in modifying these pathways?*

### Analysis

Interviews were audio-recorded and transcribed verbatim. Transcripts were checked for accuracy and names of people and specific places were removed. Data were stored and analyzed in NVivo 14.0 software (QSR International).

Qualitative study staff (ZK, UI, LP) developed a codebook (see *Supplemental Files*), using both deductive codes (derived from interview guide, theory, and literature) and inductive codes (derived from interview memos and recurring topics). The codebook was reflexively updated to reflect nuances in terms, add topics that emerged as relevant, and removal of codes that were not relevant. The study team, including ZK (lead author), HC, and UI, conducted a reflexive theoretical thematic analysis (TA), informed by Braun & Clarke’s approach, (28). TA provides a reflexive, iterative method of identifying and describing patterns in qualitative data (29). Strengths of TA include its ability to be applied to a range of different topics, research questions, and theoretical frameworks (28).

ZK and LP ***immersed themselves in the data*** through re-reading transcripts, listening to the audio recordings of interviews, reviewing interview notes, and discussing interview content with the study team. Initial ***code generation*** was driven by the interview guide (e.g., “Accepting naloxone,” “Carrying naloxone,” “Utilizing naloxone,” “naloxone opinions”). ZK, LP, and UI generated a codebook (see *Supplemental Files*) that was iteratively updated throughout coding and analysis. ZK and LP collaboratively coded the first several transcripts and subsequently coded independently, comparing every fourth transcript. Discrepancies in code definitions and application were discussed amongst the qualitative team (ZK, LP, UI).

***Theme construction*** was supported by NVivo 14.0 qualitative analysis software. ZK examined which codes were commonly grouped together and developed memos on relationships between codes. ZK then grouped code sets into preliminary themes, eliciting continuous feedback from the study team (HC, UI). ZK then ***reviewed potential themes*** and drafted diagrams illustrating how they related to one another; ***defined and named themes***, by reviewing relevant quotes within each theme and outlining how each theme contributed meaning to the research question; and ***produced an analysis report*** with accompanying data visualizations, guided by memos, diagrams, and coded transcripts. ZK elicited continuous feedback from the study team on the creation of themes and on how to refine the themes into a coherent story. Results were organized by larger thematic categories of ***accessing/accepting naloxone, carrying naloxone***, and ***administering naloxone***. Within those categories, sub-themes were mapped onto intersecting R-REF domains of ***physical, social, political, criminal-legal system, healthcare*** and ***economic environments***. Early analyses indicated that the C2H OEND intervention invited participants to become part of their local healthcare environment by supporting their capacity to help people who overdose survive, and so all themes intersect with the R-REF healthcare environment.

### Ethics

The study was approved by the University of Kentucky IRB (#52439). Prior to all interviews, oral informed consent was obtained. All recordings, transcripts, and other study materials were stored on secure, password-protected devices.

## Results

**Figure 1:**
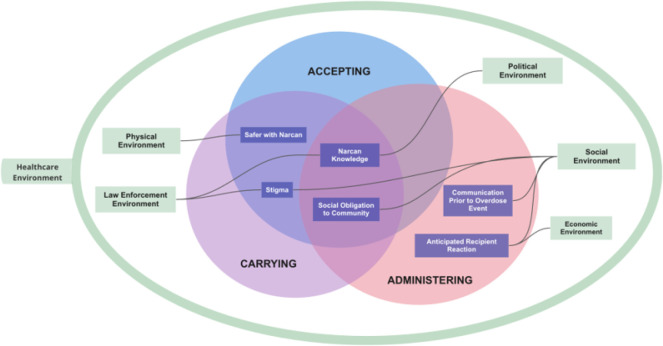
Results Visual Model

The final sample for this study included 29 participants (see Table 1). Results (see Figure 1) are organized by larger thematic categories of ***accessing/accepting naloxone, carrying naloxone*,** and ***administering naloxone***. Within those categories, sub-themes are mapped onto intersecting R-REF domains of ***physical, social, political, criminal-legal system, healthcare*** and ***economic environments***.

### 1.1 - Knowledge:

at first I was like… “ain’t no way it saves somebody’s life”

Over half of the participants (n=16) learned something new about naloxone through the intervention. Some of those 16 participants (n=5) learned about the existence and/or availability of naloxone for the first time through the C2H intervention. These participants used language like, *“I had no idea what it was”* when describing their previous knowledge of naloxone. Others were previously unaware of how to access naloxone or were hesitant to attempt to access it. For example, one participant expressed that she had heard about naloxone being offered in her community but was skeptical that it was available locally. *“I know that sometimes they would give it out [around here]... But I was like, ‘I don’t believe that. I can’t believe that.’”* Another discussed learning about the various places he could access naloxone through the intervention: *“[Through the intervention, I learned that] the [local resource center] I think has some, down there at the Health Department… There’s a few places I can get it.”*

Of the 16 participants with new naloxone knowledge, most (n=13) learned about how to administer naloxone. Participants reported that the naloxone training taught them about *“the recovery positions… putting them on their side… their mouth closed, their head tilted back”*; *“how long it takes to kick in”*, and how to time doses: *“wait two-to-three minutes between each dose”*

Through training, participants increased their confidence in naloxone administration:

“[My friend] said ‘You know you’re only supposed to give so many [Narcan doses] within so many minutes of one another?’ I said ‘Duh, I’ve been trained by [REHN].’ I said ‘I’ve watched the videos… I know how to use Narcan.”

– Male, County 2

Participants also gained confidence in naloxone’s efficacy:

**Interviewer:** Has your opinion of Narcan changed […] since joining CARE2HOPE?

**Participant:** Yeah, at first I was like, what the heck, ain’t no way it saves somebody’s life. Yeah, it does. Absolutely.

**Interviewer:** So, before you didn’t necessarily believe that [Narcan] would work?

**Participant:** No, I didn’t believe it, ain’t no way. Yeah, it works good.

– Female, County 7

Turning to the ***criminal-legal system environment,*** some participants (n=5) described learning about the legality of naloxone from the intervention, including the fact that they could not be prosecuted for possessing it. One participant said the training taught her *“that you can be arrested, you can be charged, but they can’t prosecute you and make it stick if you’re having Narcan in your pocket.”* Participants shared newfound knowledge of medical amnesty policies with their social networks. *“Everybody’s so afraid to call 911. And then I’m like, ‘Look, there’s a law passed. It’s American law… We’re getting this person help,”* another participant offered.

Participants’ receptivity to the interventions’ naloxone-related education and resources was enhanced by relationships with REHNs. Participants expressed that their social networks were limited due in part to experienced judgment and distrust. One participant recounted when she came out of prison, *“no one would trust me. Everyone waited for me to fail knowing I was going to fail.”* REHNs often met participants’ relational needs by providing nonjudgmental social support rooted in understanding of local context, shared lived experience, encouragement, validation, respect for boundaries, trust, and empathy. When describing ongoing familial conflict, one participant explained *“I feel like [my family] just doesn’t care. They don’t help me at all as far as transportation, or food, or communication, anything like that.”* When asked how her REHN had helped her navigate this period, she replied, *“He checks on us… and asks how everything is going, or if we need anything. He gives us Narcan and fentanyl test strips… Things we might need.”* For this participant, her relationship to her REHN contrasted with her relationship to her family and served as a source of emotional and instrumental support. Shared lived experience with REHNs fostered trust and supported the ability to receive naloxone.

“It’s nice talking to somebody that has also been through what I’ve been through, because [REHN]… has been a recovered addict as well. It really helped me, talking to [REHN]. [REHN] gave me Narcan…”

- Female, County 5

By providing instrumental support (naloxone distribution) paired with informational support (naloxone training, education, and policy information) and emotional support and solidarity, the intervention both altered participants’ ***healthcare environments*** and enabled them to become community overdose responders – i.e., a part of the ***healthcare environment themselves***. Almost all participants (n=27), opted to accept naloxone, indicating that individuals who receive education on naloxone are likely to accept it when offered.

Just two participants opted *not* to accept intervention-provided naloxone. They reported that they made this decision because they had access to naloxone through other channels. One explained, *“I already have Narcan… When I left the hospital, they fill your prescriptions there… and Narcan was one of them… [The hospital-provided Narcan] is in my medicine cabinet as we speak.”* For the other, the decision to accept naloxone offered through the intervention was contingent upon how much naloxone he currently possessed, and possible shortages for others in need.

“I don’t want to take something that somebody else could possibly use... If I had like 10 [Narcans] at home, that’s enough for me for the time until I use some... I don’t want to over-take something and somebody go after me and them not have it. Selfish. Because I want everybody to live.”

- Male, County 2

This participant thus invoked a community-focused mindset in which taking naloxone was contingent on his current perceived need, which can be interpreted as interplay between the ***healthcare and social environments***. However, not everyone who accepted naloxone from C2H consistently carried it or administered it, explored below.

### 2: Carrying naloxone

Approximately two-thirds (n=19) of participants opted to carry naloxone at least some of the time. The C2H OEND impacted participants’ experiences of carrying naloxone by, a) providing access to naloxone, b) increasing participant confidence in their legal right to carry naloxone, c) mitigating impact of police stigma, and d) enhancing positive feelings towards naloxone.

#### 2.1 - safer with naloxone:

“you never know when you can run into someone that is overdosing”

Participants (n=10) shared that despite potential consequences, they felt safer with naloxone than they did without it. One participant said, *“I feel safe and secure, a little bit more secure with [Narcan] on me.”* One participant explained that carrying naloxone made him feel safer considering the fentanyl influx in his community because naloxone *“helps with everything.”* When describing pervasive yet unpredictable overdose patterns in their community, another participant explained,

“You can’t ever tell in wherever you’re at, what kind of situations going on, anything can happen in the spur of a moment. And I know as long as I’ve got [Narcan] on me, if something like that ever happens around me, it could save somebody’s life.”

• Female, County 7

The local rural ***physical environment*** contributed to participants’ desire to carry naloxone. The combination of high overdose prevalence and suboptimal emergency response systems contributed to participants’ desire to carry their own naloxone rather than relying on an ambulance: *“In rural areas, ambulance doesn’t always come… time is very precious in those moments. Every second counts”* one participant said. However, there were also notable barriers that influenced participants’ decisions carry naloxone, including stigma, explored below.

#### 2.2 - Helper role:

“it feels good to have a part in something”

Regarding the ***social environment***, some participants (n=7) attributed their decision to carry naloxone to a sense of social responsibility. When discussing the factors that motivated their decision to carry naloxone, participants invoked a self-imposed social responsibility to community members – which we interpret as part of the R-REF ***social environment*** - and an enhanced sense of security associated with carrying naloxone, which we interpret as part of the ***criminal-legal system environment***. Some participants expressed that while they were no longer actively using drugs, people within their social networks were. Carrying naloxone helped these participants feel prepared to respond to overdose in their community. Participants used language like *“doing your part,”* when describing their internalized role. One participant explained: *“I can’t walk by somebody laying on the ground and not try to help […] That’s somebody’s daddy or mother or daughter or son […] I won’t walk by.”* Participants described being known within their community as someone who has naloxone:

“People would always come running to us when they would need [Narcan], when people would OD. We actually saved eight or nine, 10 lives. People would OD, we would be the ones to have the Narcan. Everywhere we go, we got Narcan. People we don’t know, we have saved their lives with Narcan […] Just because we knew we had it and they didn’t.”

-Male, County 7

One participant shared that her social role as a naloxone carrier was important because *“If I’m around [community members] and I have Narcan, they can’t misplace it or not be able to find it when they need it,”* when describing her role as someone equipped with the tools and knowledge to respond to overdose. Another participant expressed that this social role was a source of self-esteem, explaining, *“It’s like doing your part… It feels good to have a part in something.”* Two participants explained that they began carrying naloxone after witnessing a friend’s overdose. For these participants, the feeling of being unequipped to respond to these witnessed overdoses spurred a significant behavioral change. One recalled *“I didn’t have [Narcan] that night, and ever since, it’s always been with me.”*

One participant expressed that she had not experienced social obligation in the past:

I was a very careless addict. I didn’t really care. I was the type of addict on heroin that my friend would overdose in front of me and I would pick their pockets for their dope and not even call an ambulance. So I didn’t really care. I didn’t feel the need to have it.

-Female, County 7

Later in the interview, this participant went on to say that several recent life changes (e.g., moving, drug cessation, family loss) had led changes in her perceptions and behavior, and that she now does carry intervention-distributed naloxone with her.

The helper role was a critical driver for some participants’ (n=7) decision to carry naloxone. Participants said that they carried naloxone in part because they had access to it and their fellow community members often did not, amplifying their sense of social responsibility.

#### 2.3 - stigma and criminal-legal systems:

“wear a badge, they think they’re better than everyone else”

Stigma codified and perpetuated by law enforcement obstructed some participants’ comfort with carrying naloxone. Because all participants were criminal-legal system involved, relationships with law enforcement were rooted in prior conflict and experienced trauma. Several participants (n=5) described law enforcement stigma as something that had made them hesitant to carry naloxone:

There’s times I have been stopped, afraid the law would try to charge me with something... But [that was] before I got into the CARE2HOPE. Once I got into this program, it lightened my feelings up on if I got stopped, because [Narcan] actually helps people…I realized not to worry if I’ve got it, because it’s actually to help people.

–Male, County 7

In this participant’s case, the intervention mitigated the impact of police stigma. Through engaging with naloxone training, he both enhanced his positive feelings towards naloxone and reduced his fear of consequences to carrying it. Another participant explained that the desire to be prepared to respond to overdose outweighed the fear of arrest: *“I’d rather have [Narcan] on me and go to jail [than] not being able to save someone because I don’t have it.”* However, for other participants, apprehension of law enforcement stigma remained a barrier to carrying naloxone. Another participant explained, *“if I’m carrying Narcan, then that’s going to make [police] judge me or question me more and wonder why I have that if I’m not actively using.”*

Community-level stigma, defined as experienced and anticipated judgement from community members, was less salient to participants’ decisions to carry naloxone. Some reported that carrying naloxone was normalized in his community, stating, *“Everybody I know carries it, whether they’ve done a drug in their life, they still carry it… The preacher’s got some in his glove box.”* Others reported that they did not notice or care about community stigma: *“even if they did [judge me], I don’t care. That’s irrelevant to me.”* Conversely, one participant did describe experiencing stigma from a community member for carrying naloxone. *“She seen Narcan in my car and just automatically said, ‘Oh, so you’re on [drugs] now?’ To me, that was judgmental […] Just because I have [Narcan] in my car, does not mean that I’m on drugs.”*

While participants’ barriers to *carrying* naloxone largely stemmed from their ***criminal-legal system environment*,** barriers to *administering* naloxone primarily dealt with ***social environments***.

### Administering naloxone

Over half (n=16) of participants described recent experience administering C2H-provided naloxone. Fourteen participants administered C2H-provided naloxone on someone else, one participant had C2H-provided naloxone administered on her, and one participant handed someone else the naloxone to administer to another individual during an overdose event. A subgroup of participants (n=6) described administering naloxone in the past but did not have recent experience with administering C2H-provided naloxone. Another sub-group (n=6) recalled no experiences administering naloxone, before, during, or after the intervention.

Participants with recent naloxone administration experience (n=16) described a range of barriers and facilitators to administering naloxone, largely rooted in the ***social environment***. These fell into two main categories: 1) anticipation of the recipient’s reaction, and 2) prior communication between the participant and recipient about naloxone.

#### 3.1 - recipient reaction:

“now they’re sober and they’re broke”

Participants expressed that when they used naloxone to reverse someone’s overdose, they were often met with anger and frustration upon the recipient’s revival. Participants offered various explanations for these reactions, including loss of high, acute withdrawal symptoms, and frustration of having spent limited funds on drugs they can no longer feel the effects of.

Several participants had experienced overdose reversal themselves and empathized with their peers’ response. Apprehension about administering naloxone was often rooted in first-hand knowledge of overdose reversal discomfort. Participants described rapid-onset withdrawal symptoms associated with overdose reversal. One participant explained that she had been angry with people in the past for using naloxone on her because, *“You wake up sick and pissed off. You just need another shot [of heroin]… I would wake up mad as hell, then get me another shot… that way I wouldn’t be sick.”* Several participants shared that they learned about the biopsychosocial experience of overdose reversal through the intervention: *“[I learned] that Narcan puts you into straight withdrawals. That’s why you feel bad after you use it.”*

Often, recipients’ negative reactions to overdose reversal stemmed from the loss of high in addition to the physiological withdrawal symptoms described above. Nine participants spoke to recipients’ *“loss of high”* as something they considered before administering naloxone. One participant recalled that in her experience administering naloxone, the recipient wakes up *“swinging, madder than hell because I took their buzz away.”* Similarly, another participant explained that she had seen people deny Narcan because *“They didn’t want their high to go away. They were so high that it could kill them, but they didn’t want to lose their high.”*

Some participants (n=3) explained that the recipients’ reactions upon revival were driven in part by feelings of having lost or “wasted” money, a feature of the ***economic environment***. One participant explained that in her experience administering naloxone, the recipient reacts negatively because *“Now they’re sober and they’re broke… I had a man that was in full overdose one time. When he come to, he said ‘…You just caused me to waste $160.’”* Another participant recounted similar experiences:

Some of them are just like, “That’s the only money I got. If you Narcan me and I go back to being completely sober, I’m going to be mad because, pretty much, I bought those drugs for nothing. I’m not going to feel them, and I’m not going to be able to get anymore,” which, even as an addict, it’s crazy to me. I just couldn’t imagine being in that mindset and thinking that that high is more important than me waking up.

–Female, County 4

Participants often administered naloxone ***despite*** anticipated negative reactions, evidenced by the number of participants (n=14) who had recently administered intervention-provided naloxone. Participants’ rationale for administering naloxone despite negative consequences invoked some of the same sentiments that factored into decisions to ***carry*** naloxone (e.g., social obligation to community, helper role). One participant reasoned, *“You may get hit but that’s just part of it… I’m not going to lay back and watch somebody die.”* Participants were empathetic to the experience of overdose reversal, particularly when they had been on the receiving end of naloxone in the past. For another participant, the decision to administer naloxone despite potential consequences came down to considerations of the recipient’s family: *“They might be having a bad day today, but that doesn’t change the fact that they have a family that’s going to have to deal with the consequences if something happens to them.”*

Two participants said that anticipation of the recipient’s reaction made them delay naloxone administration or exhaust alternative options prior to trying naloxone. One participant explicitly stated that naloxone was a last resort: *“I’d try to save them at all costs without using Narcan if possible… I would resort to everything but [Narcan]. Use it last.”* Another participant shared that sometimes she was hesitant to administer naloxone because she feared the recipient would be upset with her. In response, she delayed administration: *“I try to wait a little longer than what I usually would […]I don’t want to wait too long, but then I have it in my head, they’re going to get mad at me… It’s nerve racking.”*

Participants overcame barriers to administering naloxone by weighing the consequences of naloxone administration (e.g., adverse recipient reaction) against the consequences of doing nothing (fatal overdose). The C2H intervention helped participants overcome barriers by providing access to naloxone (reducing scarcity, actual and perceived), increasing confidence in naloxone administration through training, and reducing fear of ***criminal-legal*** consequences through education. However, participants explained that the experience of administering naloxone, particularly to someone in one’s own social network, is wrought with complex ***social environment*** considerations. These are further illuminated in participants’ communication with social network members regarding expectations and intentions for naloxone administration.

#### 3.2 - prior communication:

“wait three minutes before you Narcan me”

Participants’ ***social environments*** featured discussions about the use of naloxone prior to overdose events involving a) the participant’s intention to administer naloxone if they observe signs of overdose, and/or b) the recipient’s preferences regarding if/when they wish to have their overdose reversed with naloxone.

Another component of these conversations was the recipient’s communicated desire, or lack thereof, to receive naloxone. Participants described receiving or giving instructions to wait for a specified benchmark or time limit before administering naloxone. *“I usually tell people, ‘if my lips ain’t blue, don’t touch me with no Narcan,’”* One participant reported that her partner told her, *‘If I go out wait at least three minutes before you Narcan me’”*

These conversations often involved one party’s intention to administer naloxone despite the other party’s objections. *“Some people say, ‘If I need [Narcan], don’t,’* one participant recounted. Her response: *“Sorry, but I’m going to.”* For some, conversations regarding intentions to administer naloxone acted as a facilitator to future naloxone administration. For others, these conversations made it more difficult to administer naloxone, particularly when the recipient expressed a strong desire not to receive naloxone. Participants shared that this barrier was often overridden by other facilitators, like social obligation to community, helper role, and positive feelings towards naloxone often developed through intervention training.

## Discussion

This study qualitatively assessed the pathways through which risk environments influence how rural PWUD accept, carry, and administer naloxone, and the role of an OEND intervention on these experiences. Analyses found that the C2H OEND intervention altered participants’ healthcare environments by providing access to naloxone, increasing participant knowledge of naloxone efficacy and administration technique, and increasing participant confidence in naloxone administration. Over half of participants gained knowledge on naloxone through the intervention related to the healthcare environment (how to access naloxone, administration technique) and political/law enforcement environment (medical amnesty policies). Through knowledge and skills gained in the intervention, participants became a part of their local healthcare environment: most participants opted to carry naloxone, citing factors related to the social environment (responsibility to community) and physical/healthcare environments (high overdose prevalence, suboptimal emergency response systems). Over half of participants had recent experience administering intervention-provided naloxone. Experiences administering naloxone to peers was largely shaped by social environment considerations (anticipated negative reaction from recipients attributable to physiological withdrawal, loss of high, and economic loss). Participants who felt strong social ties to their community often administered naloxone despite anticipated consequences.

While there is a growing body of research on naloxone experiences, our study was the first to our knowledge to qualitatively assess PWUDs’ experiences accessing, carrying, and administering naloxone in a rural area through an R-REF lens. Previous research in this area largely centered the perspective of stakeholders who do not use drugs (e.g., emergency responders, pharmacists, etc.). Studies that have centered PWUD as experts primarily take place in non-rural settings. This study built on nascent emerging research with PWUD in rural settings and applied the R-REF as a guiding framework.

### Accessing/Accepting Naloxone

Other studies conducted in non-rural settings identified **healthcare environment** factors, like PWUDs’ access to information on naloxone (23), as key drivers for accessing and accepting naloxone. Our results supported these findings in a rural area, and identified several other knowledge-based factors relevant to this particular rural population. In the rural area of the study where harm reduction efforts are still escalating, almost one-fifth of the sample learned that naloxone existed for the first time through the OEND intervention; another almost two-fifths were already familiar with naloxone but learned about naloxone’s effectiveness through the intervention. That so many participants had never heard of naloxone in 2022, when the intervention was delivered, is striking, and testifies to delayed diffusion of harm reduction interventions into rural areas compared to urban areas. Healthcare innovations are typically slower to reach rural areas; anti-PWUD stigma might further delay the diffusion of harm reduction interventions in particular. Perhaps further testifying to local anti-PWUD stigma, participants reported that REHNs were deeply respectful of them, and thus could be a trusted source of information about naloxone. Participants described that their trust in REHNs was rooted in REHNs’ understanding of local context, shared lived experience, encouragement, validation, respect for boundaries, and empathy. Some participants explained that conversations with their REHN were very different than stigma-laden interactions with other healthcare providers and community members.

For many participants, decisions to accept naloxone were predicated not only on understanding what naloxone was and learning about its effectiveness, but also on learning about protections offered by the state’s medical amnesty/Good Samaritan policies. These criminal-legal system/political considerations were especially relevant because our participants were criminal-legal involved and distrusted law enforcement. Learning about their legal rights helped participants feel more confident carrying and administering naloxone. However, the intervention did not impact policy or conduct outreach and education to law enforcement or other criminal-legal system representatives. Therefore, the intervention’s impact was limited to participants’ knowledge and confidence surrounding naloxone.

### Carrying Naloxone

Previous studies conducted in both urban and rural areas identified feelings of purpose and empowerment (40, 41, 42) as facilitators to *administering* naloxone. Few studies explored these feelings in relation to *carrying* naloxone. Our findings partially aligned with this prior work; participants in this rural area did not highlight empowerment, but instead emphasized a sense of social responsibility to their community. These feelings factored strongly into decisions to carry and to administer naloxone. Social responsibility plays a critical role in rural communities where social networks can be integral to survival (32). The theme of social responsibility emerged in various contexts, including community leaders’ support of naloxone with one participant noting *“even the preacher’s got some [naloxone] in his glove box.”* This dynamic represents one end of the participant experience spectrum, with other participants in our sample describing pervasive community stigma towards harm reduction strategies. Our findings in this area speak to an evolving cultural landscape around naloxone and other harm reduction strategies where attitudes and norms appear to vary by community.

Our findings identified that participants’ rural physical environments often facilitated their desire to carry naloxone, highlighting the high prevalence of overdose in their community combined with slow emergency response systems in a geographically dispersed area with few roads per acre. One participant reasoned, *“In rural areas, ambulance doesn’t always come… Time is very precious in those moments. Every second counts”* when explaining why they chose to carry naloxone. This area of findings highlights a key feature of our study environment and other rural areas where populations are denser and thus ambulance travel times far shorter (33).

### Administering Naloxone

Our findings highlight fear of disrupting someone’s high and fear of imposed economic loss as paired barriers to naloxone administration decisions. Previous studies in urban areas identified fear of recipient reaction (25) and fear of disrupting someone’s high (34) as key social barriers to naloxone administration. Our results supported these findings with the caveat that for most participants, this barrier did not prevent them from naloxone administration. It did however, complicate and sometimes delay the administration process. We could find no prior research in urban settings that described conversations among PWUD about whether/when to administer naloxone. In this rural setting, where social bonds were strong and networks quite dense (35), participants told us conversations about naloxone with peers prior to overdose events could act as barriers or facilitators to future naloxone administration. For some participants, the opportunity to clearly communicate intention to administer naloxone bolstered preparedness and confidence. For others, hearing the other party’s desire to avoid naloxone complicated future administration decisions. We also found that economic loss (e.g.. *“Now they’re sober and they’re broke… I had a man that was in full overdose one time. When he come to, he said ‘…You just caused me to waste $160.”)* heavily factored into naloxone administration considerations. This finding speaks to the influence of the economic environment in naloxone decision-making, which plays a pivotal role in economically disenfranchised communities. Our participants’ emphasis on economic loss related to naloxone administration is relevant and meaningful, given the economic-historical context of rural Appalachian Kentucky, our study setting.

### Strengths & Limitations

Results of this study fill a gap in the literature regarding naloxone experiences of PWUD in rural Appalachian communities. Furthermore, our study explored the ways that PWUD discuss naloxone with their social networks, before, during, and after overdose events. This focus area yielded rich data, providing meaningful context to the existing body of literature. The application of the R-REF as the guiding framework for our study is a key strength of this study, missing from other studies that explore naloxone experiences in rural environments. This framework helps us to contextualize findings in relation to a long-standing body of scholarly work (7, 10, 14) and helps to identify environmental drivers of behavior that are unique or more relevant to rural settings.

Among the limitations of this study is missing perspectives. Our sample was skewed towards participants with sustained engagement in the intervention. We lack perspectives of people who were re-incarcerated or otherwise disengaged from the program during our recruitment period. Furthermore, our sample was largely comprised of white participants, meaning that we lack important perspectives of rural people of color.

During data collection, some interviews were impacted by poor cellular/internet connection. While the researchers took steps to mitigate disruption (e.g., arranging office space/computers for participants to use, supplementing transcripts with detailed notes), transcripts still reflect some inaudible moments.

Another limitation is that naloxone experiences were not the sole focus of the interview guide. Furthermore, the naloxone questions were placed towards the end of the guide. While this structure allowed the interviewer to build trust and rapport with participants before discussing naloxone experiences, it also increased risk of participant fatigue.

Regarding positionality, the primary interviewer is not from a rural Appalachian community. While the interviewer strived to build rapport and avoid stigmatizing language, she may have been perceived as an outsider by participants. However, having third party distinct from the C2H REHNS conducting interviews was a methodological strength as it enabled participants to openly share their experiences.

## Conclusion

Factors that influence rural PWUDs’ decisions to accept, carry, and administer naloxone are complex and touch upon all domains of their rural risk environment. Our sample included participants who had never heard of naloxone or had gaps in their understanding of its effectiveness and/or how to administer it. The C2H intervention altered participants’ healthcare environment by providing education on naloxone’s existence, effectiveness, and administration technique. Participants’ trust in REHNs (driven by REHNs’ knowledge of the community and non-stigmatizing approach) allowed participants to be receptive to new information about naloxone. Participants’ physical environments, characterized by slow emergency response systems, high prevalence of overdose, and presence of fentanyl drive decisions to carry naloxone despite anticipated and realized consequences (e.g., police harassment). Participants’ decisions to administer naloxone are sometimes complicated by social environment considerations. When PWUD administer naloxone to a network member, they are often acting against the recipient’s communicated desires and are sometimes met with anger. Although many rural PWUD administer naloxone despite social consequences, participants report that the event can be emotionally difficult, necessitating multipronged means of support.

Findings highlight need for more outreach to rural PWUD on local policy that impacts them; tailored strategies to help PWUD engage in productive dialogue with peers about naloxone and navigate interpersonal conflict associated with overdose reversal; and opportunities for rural PWUD to formally participate in emergency response systems as peer overdose responders.

## Figures and Tables

**Table 1:** Description of Sample

N=29	Frequency	Percentage
**Gender**		
*male*	11	37.93
*female*	18	62.07
**Education**		
*Less than high school*	10	34.48
*High school diploma or GED*	10	34.48
*Some college*	8	27.59
*Associates degree/trade or technical school*	1	3.45
**Race**		
*White*	28	96.55
*Other*	1	3.45
**Current drug of choice for getting high**		
*Heroin*	11	37.93
*Opiate painkillers*	2	6.90
*Buprenorphine*	2	6.90
*Benzodiazepines*	1	3.45
*Methamphetamine*	12	41.38
*Gabapentin*	1	3.45
**Ever used naloxone on someone to reverse overdose**		
*No*	9	31.03
*Yes*	17	58.62
*Did not answer*	3	10.34
***Baseline:* Have naloxone with them or at home at any point in past 90 days**		
*No*	1	3.45
*Yes*	19	65.52
*Did not answer*	9	31.03
***3-months:* Have naloxone with them or at home at any point in past 90 days**		
*No*	5	22.73
*Yes*	17	77.27

**Table 2:** Interview Guide naloxone/Narcan Questions

Question	Probes	
**When you were offered Narcan, did you accept it?**	*If not:* Why not?	
**Was this your first time you received Narcan?**	*If first time:* Had you heard of Narcan before? → Did you know where to get it?	*If received Narcan before C2H:* Where had you gotten Narcan in the past? → How often had you used it before C2H? → Did this experience make you more/less likely to carry Narcan in the future? → Can you tell me more about that?
**Did the C2H training teach you anything new about Narcan**	What did you learn about Narcan? → Did you feel like you knew what to do with Narcan if you’d needed to administer it? → What more would you have liked to learn from us?	
**Do you carry the Narcan with you? Why/why not?**	Is there anything that makes you nervous about carrying Narcan with you? → Stigma/judgement? → Criminal justice involvement?	
**Have you used the Narcan we gave you?**	*If not:* Why not?	*If used:* Please tell me more about your experience → Who was overdosing? → How did you know this person? → How did you know they were overdosing? → What did you do? → Did you or anyone call 911? → Did police arrest or charge anyone? → Were you nervous about being arrested or charged?
**Have you had Narcan used on you?**	*If yes:* How did that experience change your opinion of Narcan?	
**How did having Narcan or having the training change your behavior?**	How did it change how you use drugs? → Did you share information about overdosing that you learned from us with others? → Did you give away/sell any of the Narcan to others?	
**Did you ever worry that someone would be upset if you administered Narcan on them?**	*If yes:* Can you tell me more about that? → Have you had past experiences where someone got upset with you for using Narcan → What did you do?	
**Has your opinion of Narcan changed at all since you started the intervention?**	How?	

**Table 3:** Sub-set of Codebook Pertaining to naloxone/Narcan

Code name	Definition	Example
*Receiving Narcan*	Refers to participant’s experience receiving Narcan from C2H staff	“It really helped me, talking to [them]. [They] gave me Narcan.”
*Carrying Narcan*	Refers to participants’ storage of Narcan including whether they opt to carry Narcan on their person	“I haven’t had to use it yet. It’s in the cabinet at the house that I usually stay at. I can’t use it because I’m allergic to it.”
*Utilizing Narcan*	Refers to participant’s experience utilizing Narcan. If highlighting non-C2H Narcan, cross-code with “before C2H”	“I would have lost those two a couple of times because they were[...] Just a squirt to get them to wake up, so that video that I watched up there probably saved their lives.”
*Narcan used on participant*	Refers to participant’s experience having Narcan used on them	“It wasn’t that I was upset that I was being resuscitated. It was the feeling that the Narcan gave me. It made my whole body go ice cold, and I started shaking. Because Narcan reverses the effects of the heroin, which made you go in sudden, rapid withdrawal times 50.”
*Narcan perceptions/feelings*	Refers to participant’s feelings, opinions, and perceptions of Narcan	“Even if you don’t need it, it’s a good, people see it as a good thing to have.”
*Narcan knowledge, existence*	Refers to participant’s prior knowledge or learning of what Narcan is	“I didn’t know what it was”
*Narcan knowledge, effectiveness*	Refers to participant’s prior knowledge or learning of Narcan’s effectiveness in reversing overdose	“At first I was like... ain’t no way it saves somebody’s life. Yeah, it does. It works good”
*Narcan knowledge, amnesty*	Refers to participant’s knowledge or learning of Good Samaritan or medical amnesty laws/policies	“you can be arrested, you can be charged, but they can’t prosecute you and make it stick if you’re having Narcan in your pocket.”
*Narcan, social obligation*	Refers to participant’s self-imposed social role as a community helper, regarding Narcan	*“I can’t walk by somebody laying on the ground and not try to help... That’s somebody’s daddy or mother or daughter or son... I won’t walk by.”*
*Narcan, “safer”*	Refers to participant feeling “safer with Narcan” than without it	“I feel a lot safer with Narcan”
*Narcan, unpredictable nature of overdose*	Refers to participants perception of community overdose as unpredictable or chaotic, regarding Narcan	“I know when I have [Narcan] on me, because you can’t ever tell in wherever you’re at, what kind of situations going on, anything can happen in the spur of a moment. And I know as long as I’ve got that on me, if something like that ever happens around me, it could save somebody’s life.”
*Narcan, stigma*	Refers to participants perceived stigma from law enforcement and/or community members regarding Narcan	“if I’m carrying Narcan, then that’s going to make [police] judge me or question me more and wonder why I have that.”
*Narcan, recipient reaction*	Refers to participant recounting or anticipating instances a person’s physiological or emotional reaction to receiving Narcan	“He says that you feel rough after you get Narcan...for a day or two.”
*Narcan, loss of high*	Refers specifically to a Narcan recipient being frustrated that they can no longer feel effect of drugs	“They didn’t want their high to go away. They were so high that it could kill them, but they didn’t want to lose their high.”
*Narcan, economic loss*	Refers specifically to Narcan recipient being frustrated that they spent limited funds on drugs they can no longer feel the effect of	“You don’t want to lose that feeling that you paid for”
*Narcan, prior communication*	Refers to participant recounting conversations they have had with network members regarding Narcan, prior to overdose events	“‘If you nod out, and if you don’t respond to me. I will Narcan you.’ Even before they [use drugs], I’m like, ‘I do have Narcan. I will Narcan you.’”

## Data Availability

The interview transcripts are not publicly available for confidentiality reasons.

## Abbreviations

C2H: CARE2HOPE
OEND: Overdose Education & Naloxone Distribution
PWUD: People who use drugs
R-REF: Rural Risk Environment Framework
